# Instant messaging-based digital health interventions for diabetes management: a domain-structured systematic review and meta-analysis of randomized controlled trials

**DOI:** 10.3389/fpubh.2026.1780625

**Published:** 2026-03-09

**Authors:** Shan Chen, Emma Mirza Wati Mohamad, Arina Anis Azlan, Xixi Zhao

**Affiliations:** Faculty of Social Sciences and Humanities, Universiti Kebangsaan Malaysia (UKM), Bangi, Selangor, Malaysia

**Keywords:** diabetes, digital health, instant messaging, meta-analysis, randomized controlled trials, self-management, social media

## Abstract

**Background:**

Instant messaging delivered through social platforms is increasingly used to support diabetes self-management. However, evidence remains difficult to interpret because trials vary widely in platform choice, intervention design, outcome constructs, and measurement instruments.

**Objective:**

To synthesize the effects of instant-messaging interventions for diabetes across pre-specified outcome domains, and to explore whether platform type, follow-up duration, and study size help explain variation in effect estimates.

**Methods:**

We searched seven databases (2010–2025) for randomized controlled trials in which diabetes interventions were primarily delivered via instant-messaging/social platforms. Outcomes were organized a priori into six domains: health behaviors, diabetes knowledge, attitudes/self-efficacy, glycemic outcomes, other clinical outcomes, and diabetes-related complications. Continuous outcomes were pooled as standardized mean differences (SMDs) and binary outcomes as risk ratios (RRs) using random-effects models (REML). To improve interpretability, we prioritized domain-level synthesis and performed platform-stratified pooling only when at least three effect sizes were available within a given domain. Heterogeneity was summarized using τ^2^ and I^2^. Robustness was assessed using leave-one-out analyses; small-study effects were evaluated using funnel plots and Egger's test when ≥10 studies contributed to an analysis. Exploratory meta-regression examined follow-up duration and ln(sample size).

**Results:**

Twenty-three trials contributed 236 effect estimates. Overall pooled effects across all continuous and binary outcomes were close to null and statistically non-significant, with substantial heterogeneity. Domain-specific synthesis showed clearer patterns: diabetes knowledge demonstrated the largest pooled improvement (SMD = 1.065, 95% CI 0.185–1.944), glycemic outcomes improved on continuous measures (SMD = −0.519, 95% CI −0.719 to −0.319), and behavioral outcomes showed a small but significant benefit (SMD = 0.359, 95% CI 0.010–0.709). Attitudes/self-efficacy and other clinical outcomes were more heterogeneous and did not show clear pooled benefits. For complications (binary outcomes), the pooled estimate suggested a potential reduction in risk (RR = 0.67, 95% CI 0.44–1.00) based on three studies and should be interpreted cautiously. Platform-overview pooling of continuous outcomes suggested variability across platforms, with more consistently positive pooled effects for Facebook Messenger-based interventions than for WhatsApp or WeChat; however, platform-by-domain pooling was often not estimable because many platform–domain combinations contributed fewer than three effect sizes. Meta-regression did not identify a clear linear association of follow-up duration or ln(sample size) with effect size, and explained little heterogeneity.

**Conclusions:**

Instant-messaging interventions for diabetes do not yield a clearly favorable overall pooled effect, but they show credible benefits for behavioral outcomes and selected clinical endpoints. Variation in effects appears more consistent with differences in intervention design and implementation than with platform labels alone. Future trials should report intervention components and maintenance strategies in greater detail and evaluate interactive, care-integrated messaging models.

**Systematic review registration:**

https://www.crd.york.ac.uk/PROSPERO/view/CRD420251079157, identifier: CRD420251079157.

## 1 Introduction

Diabetes is one of the most pressing global chronic health challenges. The International Diabetes Federation estimates that more than 500 million people worldwide are currently living with diabetes, a figure projected to surpass 600 million by 2030 (1). Beyond elevating the risk of cardiovascular disease, kidney failure, and vision loss, diabetes imposes a substantial economic burden on health systems and societies (2). Despite advances in pharmacotherapy, achieving and sustaining optimal glycemic control remains difficult because long-term lifestyle modification and self-management are hard to maintain (3). These realities underscore the need for innovative, scalable approaches that strengthen education, self-care, and day-to-day behavior change.

The rapid expansion of digital health has created new opportunities for chronic disease management. Among available tools, social media platforms have attracted growing attention as channels for health education, peer support, and behavior change. Platforms such as Facebook, WeChat, WhatsApp, LINE, and Telegram enable interactive communication among patients, health care professionals, and communities, offering advantages of accessibility, scalability, and cost-effectiveness (4–6). Evidence from individual randomized controlled trials (RCTs) suggests that social-media–based interventions may improve self-care behaviors and diabetes knowledge, and in some instances glycemic outcomes (7, 8). However, findings remain inconsistent across studies, with several reporting negligible or even adverse effects. Differences in platform affordances, intervention dose and duration, measurement choices, and—critically—the outcome level being targeted (upstream knowledge, attitudes, and behaviors vs. downstream glycemic and clinical endpoints) likely contribute to these discrepancies.

In this review, we focus specifically on instant-messaging-based interventions delivered via social media or mobile messaging platforms. “Instant-messaging interventions” are defined as synchronous or asynchronous message-based communication delivered through social-media or mobile messaging platforms (eg., WhatsApp, WeChat, LINE, Messenger), excluding SMS-only systems, standalone apps, or non-interactive web portals.

Consensus has not been reached regarding the overall effectiveness of social-media–based interventions in diabetes care, nor is it clear whether specific platforms consistently outperform others (9, 10). Prior reviews frequently pooled heterogeneous “digital” interventions without distinguishing social media platforms, or focused narrowly on single endpoints such as glycemic control, limiting interpretability (11–15). Moreover, few syntheses have adopted a domain-explicit framework that separates upstream behavior-change constructs from downstream biomedical outcomes, or have provided platform-level comparisons using pre-specified moderator analyses. The role of small-study and publication biases has also been unevenly assessed.

Specifically, this study intends to answer the following research questions:

1. Do instant-messaging/social media–based interventions yield statistically significant pooled effects within each pre-specified outcome domain?
2. Do pooled effects differ by platform and by the six pre-specified outcome domains (behavior, knowledge, attitude/self-efficacy, other clinical outcomes, complications, and glycemic outcomes)?
3. Are study-level characteristics [follow-up duration and log-transformed sample size, ln(n)] associated with effect sizes in exploratory meta-regression analyses, and do they explain any between-study heterogeneity?
4. Are the findings robust to sensitivity analyses (leave-one-out), and is there evidence of small-study effects/publication bias (funnel plots and Egger's test when applicable)?

To answer the above research questions, we conducted a systematic review and meta-analysis in accordance with the PRISMA reporting norms based on the pre-established research plan. Report the combined effects in the outcome domain and at the social media platform level (only when the included effect size is ≥3), quantitatively assess the heterogeneity between studies, and conduct targeted sensitivity analyses and small-sample effect/publication bias assessments (for details, see Section 2).

## 2 Research design and methods

### 2.1 Protocol and registration

This review follows the PRISMA 2020 guidelines and was prospectively registered in PROSPERO (CRD420251079157). Any protocol deviations were documented in the registry.

### 2.2 Data sources and search strategy

We searched seven databases: PubMed, Embase, Web of Science, ProQuest, APA PsycInfo, CINAHL, and Scopus. The time window was 20 June 2010 to 20 June 2025, with no language restrictions. Search strategies combined controlled vocabulary and free-text terms for diabetes, social-media platforms (eg., Facebook, WeChat, WhatsApp, LINE, Telegram, KakaoTalk, Naver Band), and randomized trials. Full search strings for each database are provided in Table 1. Reference lists of included studies and relevant reviews were screened to identify additional records.

**Table 1:** Characteristics of the included randomized controlled trials.

**Study**	**Platform**	**Duration (weeks)**	**Comparator**	**Intervention content summary**	**Main outcomes**
Alghafri et al. (37)	WhatsApp	24	Usual care	Community walking groups + WhatsApp prompts; clinic coaching; peer support.	Behavior ↑; some glycemic improvement at 6 months.
Bender et al. (38)	Facebook	24	Wait-list/usual care	3-month mobile lifestyle coaching (phase 1) with app/SMS; behavior goals; then 3-month follow-up.	PA and diet ↑ at 3 months; small HbA1c change by 6 months.
Xu et al. (39)	WeChat	24	Printed handbook education	6-month WeChat modules on diet & PA with tailored feedback; group chat support.	Diet & PA scores ↑; modest HbA1c reduction at 6 months.
Shi et al. (40)	WeChat	12	Usual care/education	Culturally adapted messaging/community content; scheduled posts and feedback.	Knowledge & behavior ↑; early glycemic signals.
Sutthiworapon et al. (41)	LINE	4	Pamphlet (usual education)	Three weekly LINE infographics + 1-week post assessment; visual education.	Knowledge ↑ immediately; clinical outcomes unchanged at 4 weeks.
Safdari et al. (42)	Telegram	12	Usual clinic education	Telegram-based diabetes education and reminders; weekly sessions.	Knowledge & self-care ↑; short follow-up—limited HbA1c change.
Kim and Utz (43)	Naver Band	9	Usual care	SNS-based diabetes self-management with weekly posts and peer interaction; nurse moderation.	Knowledge, self-efficacy ↑; HbA1c unchanged within 9 weeks.
Kim et al. (44)	KakaoTalk	12	Usual care	Automated KakaoTalk/SNS push with educational content and reminders; clinician oversight.	Adherence/behavior ↑; glycemic movement at ≥12 months in follow-up.
Alanzi et al. (45)	WhatsApp	6	Usual care	WhatsApp-delivered self-care lessons and Q&A; nurse moderation.	Self-care ↑; HbA1c modest change at 6 weeks (to verify).
Petrovski and Zivkovic (46)	Facebook	52	Usual care	Facebook group education, peer modeling, and Q&A; clinic linkage.	Sustained engagement; HbA1c/clinical signals with prolonged use.
Valença et al. (47)	WhatsApp	52	Usual care	Social media channels delivering nutrition guidance on low-GI diet; cooking demos/infographics.	Diet quality ↑; weight/glycemic movement over >12 months (to verify).
Leong et al. (48)	LINE	16	Usual care/education	Social-media–delivered patient education to enhance diabetes literacy; structured modules.	Literacy ↑; behavior ↑; limited short-term glycemic change.
Xia et al. (49)	WeChat	24	Usual care	Web-based TangPlan with WeChat messaging; care-plan dashboards; periodic feedback.	Glycemic and care-process improvements at 24 weeks.
Cheng et al. (50)	WeChat	12	Usual care	WeChat multimedia extended nursing after discharge; scheduled check-ins and Q&A.	Knowledge/behavior ↑; early glycemic trends.
Yu et al. (56)	WeChat	12	Usual care	Home-based web follow-up program; periodic contacts; behavior reinforcement.	Knowledge/behavior ↑; FBG/HbA1c small change.
Feng et al. (57)	WeChat	12	Usual care	WeChat group management with short-term observation; team feedback.	Behavior ↑; short-term glycemic neutral.
Shi (58)	WeChat	12	Usual care	WeChat platform extended nursing for young/middle-aged adults; fixed-interval follow-ups.	Knowledge/behavior ↑; short-term glycemic neutral.
Sadeghian et al. (59)	WhatsApp	12	Usual care	Nurse-led social media education with structured weekly topics and coaching.	Knowledge/behavior ↑; early glycemic signals.
Chen et al. (51)	WeChat	24	Usual care	Community-based online low-GI diet + lifestyle advice; group support online.	Weight/glycemic improvements with adherence.
Duan et al. (52)	WeChat	24	Usual care	Family-engaged eHealth program using social and messaging tools; parental/family support.	Behavior ↑; HbA1c improvement at 6 months.
Dong et al. (53)	WeChat	52	Usual care	WeChat applet with diet/PA self-monitoring, tailored feedback; group chat.	Behavior ↑; HbA1c decrease by 12 months.
Salarkarimi et al. (54)	WhatsApp	24	Usual care	WhatsApp-based self-care curriculum; scheduled lessons and adherence support.	Self-care ↑; HbA1c small decrease at 24 weeks.
Alanzi (55)	WhatsApp	6	Usual care	Education app/WhatsApp variant; structured lessons (to verify).	Education outcomes ↑.

Data are summarized for all randomized controlled trials included in the systematic review and meta-analysis.

### 2.3 Eligibility criteria

Inclusion criteria were as follows: (i) adults or children with type 1 or type 2 diabetes, or high-risk populations (prediabetes, overweight/obesity, metabolic syndrome); (ii) an intervention primarily delivered via a social-media or mobile messaging platform; (iii) a comparator including usual care, minimal intervention, or other non-digital controls; (iv) at least one pre-specified outcome domain (behavior, knowledge, attitude, other clinical outcomes, complications, or glycemic outcomes); and (v) randomized controlled trial (individual or cluster-randomized design). Exclusion criteria were: non-RCT designs; protocols, reviews, or editorials; conference abstracts without extractable data; interventions not primarily using social media or instant messaging; and outcomes not convertible to standardized mean differences (SMDs) or log risk ratios (logRRs). The characteristics of the included trials are presented in Table 1.

### 2.4 Study selection

**Figure 1:**
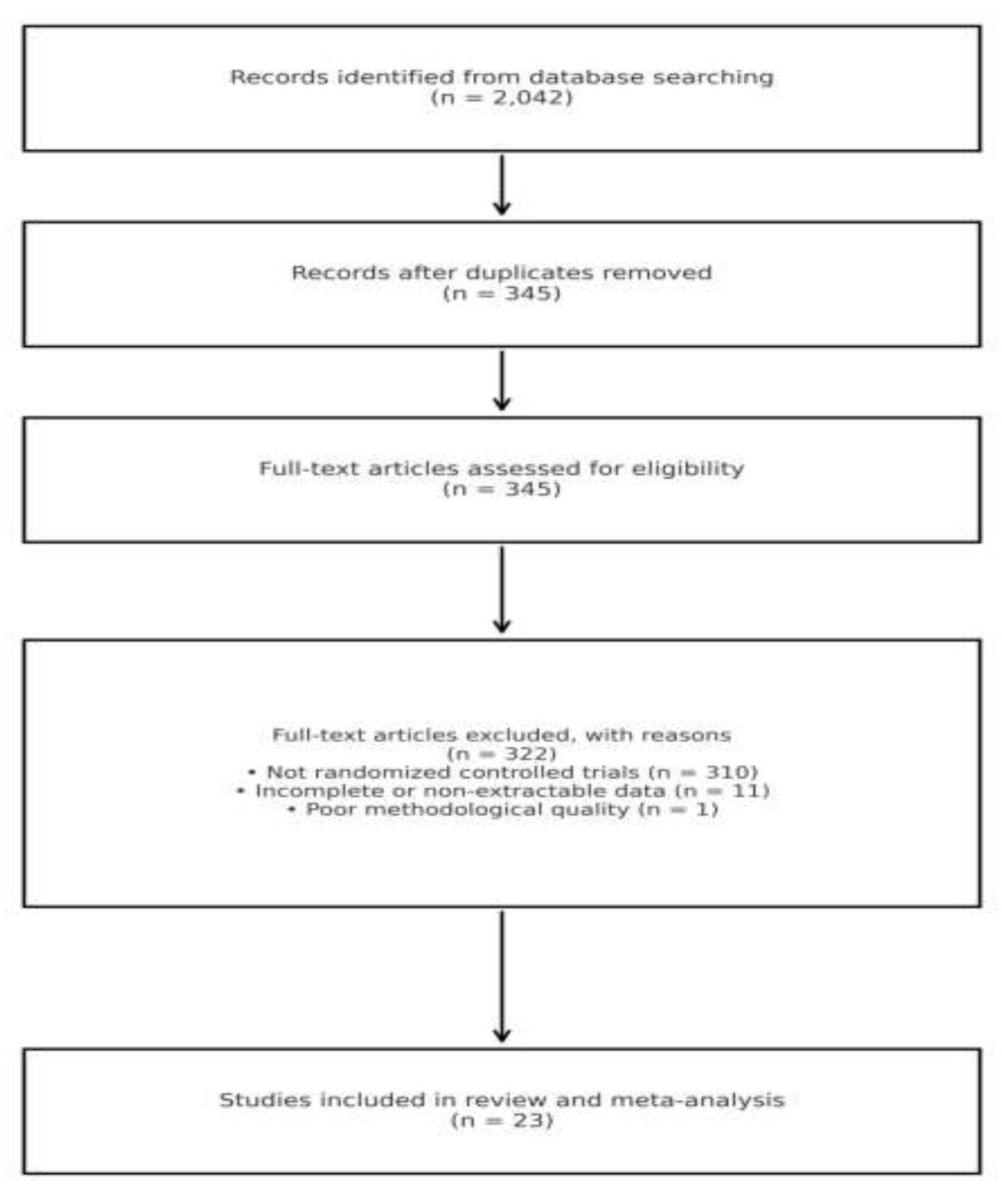
PRISMA 2020 flow diagram for study identification and selection.

Two reviewers independently screened titles and abstracts, followed by full-text screening of potentially eligible articles; conflicts were resolved by discussion. Reasons for full-text exclusion were recorded (eg., inadequate outcome data, mixed chronic-disease samples without separable diabetes data). A total of 2,042 records were identified from the database searches. After removal of duplicates, 345 records remained for title and abstract screening. All 345 full-text articles were assessed for eligibility; 322 were excluded (310 were not randomized controlled trials, 11 had incomplete or non-extractable data, and 1 was judged to be of poor methodological quality), leaving 23 studies for inclusion in the review and meta-analysis. The selection process is summarized in the PRISMA 2020 flow diagram (Figure 1). Non-randomized studies were excluded to reduce the risk of selection bias and confounding, and to ensure methodological consistency across included studies. Given the substantial heterogeneity in intervention intensity, messaging frequency, and outcome assessment in real-world studies, we prioritized randomized controlled trials to strengthen internal validity and causal inference in pooled effect estimates (16, 17).

### 2.5 Risk of bias assessment

Risk of bias was evaluated using the Cochrane Risk of Bias 2 (RoB 2) tool for randomized trials (18). Two reviewers independently assessed each study across the five RoB 2 domains (randomization process, deviations from intended interventions, missing outcome data, outcome measurement, and selection of the reported result). Disagreements were resolved through discussion with a third reviewer who acted as adjudicator.

Risk-of-bias judgments were not used as formal stratification variables in meta-analytic models because of the limited number of studies within individual subgroups (19). Instead, we incorporated study quality into robustness assessments through multiple sensitivity analyses. Specifically, we conducted leave-one-out (LOO) analyses for all models and performed additional influence analyses in which the 10 most influential studies were excluded. These procedures were used to ensure that high-risk or influential trials did not disproportionately drive the pooled estimates.

### 2.6 Data synthesis

According to the pre-specified protocol, we included original studies evaluating social-media or instant-messaging–based interventions for diabetes management, provided that outcome data were directly meta-analyzable or could be converted into a common effect-size metric using standard formulas. Given substantial variation in measurement instruments and scales across studies, continuous outcomes were primarily synthesized using the standardized mean difference (SMD; Cohen's d). Effect directions were coded so that positive values consistently indicated results favorable to the intervention. When an outcome was measured on an identical scale across studies, mean differences (MDs) were used for descriptive comparison; however, primary pooling for continuous outcomes was based on SMDs to ensure comparability.

For continuous outcomes, we extracted post-intervention group means and standard deviations (or, when reported, mean changes with corresponding standard deviations/standard errors) at the latest eligible time point to avoid double counting across multiple follow-up assessments. When standard errors were not directly reported, they were derived from confidence intervals, *P-values*, or raw data (sample sizes and standard deviations) using conventional formulas. Effect estimates for which the standard error could not be derived were excluded, and reasons for exclusion were documented in the Supplementary material.

Consistent with guidance from the Cochrane Handbook (Chapter 3) regarding multiplicity and statistical dependence, when a trial reported multiple correlated glycemic outcomes, we pre-specified a single-outcome decision rule and extracted at most one glycemic effect size per trial for pooled analysis (16). Specifically, we prioritized HbA1c, followed by fasting blood glucose (FBG/FPG) and 2-h postprandial glucose (2hPG) (HbA1c > FBG/FPG > 2hPG).

Binary outcomes (e.g., diabetes-related complications) were synthesized using risk ratios (RRs). We extracted 2 × 2 event count data to compute RRs and pooled log-transformed RRs under random-effects models. Pooled estimates were back-transformed and presented as RRs with 95% confidence intervals.

To further limit within-trial dependence arising from multiple outcomes within the same domain, we applied the rule that each trial contributed at most one effect size per outcome domain. Selection followed pre-specified priority rules for representative indicators: within the “other clinical/medical outcomes” domain (ClinicalOther), BMI was prioritized, followed by weight, blood pressure, and lipid-related indices; within the behavior domain, validated composite self-management measures (e.g., total self-care or total SDSCA scores) were prioritized, and domain-specific indicators (e.g., diet or exercise) were used when composite measures were unavailable. These procedures were implemented to enhance the interpretability and robustness of pooled estimates while minimizing statistical dependence.

### 2.7 Stratified analyses

Building on the pre-specified six-domain framework, we evaluated heterogeneity using two sets of stratified random-effects meta-analyses: platform-level stratification and outcome-domain stratification. For platform-level analyses, we computed separate random-effects pooled estimates for major platforms (WhatsApp, WeChat, LINE, Facebook Messenger, Telegram, KakaoTalk, and Naver Band). Each trial was assigned to a single dominant delivery platform. When interventions spanned multiple platforms, a trial was included in platform-stratified analyses only if a primary delivery platform was explicitly identified; trials without a clearly dominant platform were excluded from platform-level stratification.

To avoid unstable estimates driven by sparse evidence, pooled results were reported only when the number of available effect sizes in a given stratum reached k ≥ 3 (k defined as the number of effect sizes, not the number of trials). Robustness was evaluated using leave-one-out (LOO) sensitivity analyses and by inspecting prediction intervals where applicable.

To facilitate cross-trial synthesis and maintain consistency with the theoretical framework, all outcomes were pre-divided into six domains: (1) glycemic control, (2) other clinical/medical outcomes, (3) diabetes knowledge (4) health behaviors, (5) attitudes/self-efficacy, (6) diabetes-related complications (20–25). This taxonomy was theory-driven, drawing on the Knowledge–Attitude–Practice (KAP) model and aligned with prior diabetes outcome frameworks, enabling clearer distinction between upstream outcomes (knowledge, attitudes, behaviors) and downstream outcomes (glycemic control, other clinical indices, complications) (20). For continuous outcomes, effect sizes were expressed as SMDs with directions aligned so that larger values reflected improvement; for dichotomous complications, effect sizes were synthesized as logRRs and presented as RRs after back-transformation. Within each domain, continuous and binary outcomes were pooled separately under random-effects models (26, 27).

In addition to domain-specific platform stratification, we conducted a descriptive “platform overview” analysis, in which all eligible continuous effect sizes (SMDs) within each platform were pooled without separating outcome domains. This overview was intended to summarize the overall distribution and heterogeneity of continuous effects at the platform level, rather than to support domain-specific inference. To minimize interpretive bias introduced by cross-domain aggregation, primary conclusions were based on domain-stratified results, and the platform overview was used only as a supplementary descriptive summary (28, 29). As with other stratified analyses, results were reported only when k ≥ 3, and LOO sensitivity analyses were conducted for all eligible strata.

### 2.8 Random-effects model and heterogeneity

All meta-analyses were conducted using random-effects models to account for potential variation in true effects across studies. Between-study variance (τ^2^) was estimated using restricted maximum likelihood (REML). Statistical heterogeneity was quantified using τ^2^ and I^2^ and tested using Cochran's Q statistic. I^2^ represents the proportion of total variability attributable to between-study heterogeneity rather than sampling error, while the Q test evaluates whether observed dispersion exceeds that expected by chance (30).

Given substantial clinical and methodological heterogeneity across trials—including differences in intervention platforms, outcome constructs, and measurement instruments—our primary synthesis did not rely on a single global pooled estimate. Instead, following a pre-specified strategy, we conducted stratified meta-analyses by platform and by outcome domain, estimating pooled effects and heterogeneity metrics within each stratum. To reduce the risk of unstable or misleading estimates in sparse strata, pooled results were reported only when at least three effect sizes were available (*k* ≥ 3) (30, 31).

This stratified approach minimizes interpretive bias that can arise from inappropriate aggregation across highly heterogeneous interventions and outcomes, and it provides a clearer, decision-relevant characterization of how effects vary by platform and outcome domain, thereby supporting subsequent sensitivity and bias assessments.

### 2.9 Assessment of correlation between follow-up duration and study size

To assess whether there is a systematic association between the follow-up duration and the study size, and to eliminate potential confounding at the study design level, we further fitted a linear regression model at the study level with the natural logarithm of the sample size ln(n) as the dependent variable and the follow-up duration (measured in weeks) as the independent variable, and used robust standard errors for estimation. This analysis aims to examine whether the follow-up time is systematically correlated with the sample size at the study design level, in order to avoid explanatory bias caused by the correlation between dependent variables in subsequent meta-regression (32).

### 2.10 Sensitivity and robustness analyses

To assess robustness, we performed leave-one-out (LOO) sensitivity analyses for each reportable meta-analytic model. Specifically, within each pre-specified analytic unit (stratified by outcome domain or by platform) that met the reporting threshold of at least three effect sizes (*k* ≥ 3), we iteratively excluded one study at a time and re-fitted the random-effects model. This procedure was repeated until every study in the stratum had been omitted once. For each iteration, we recorded the re-estimated pooled effect and its 95% confidence interval, as well as heterogeneity metrics (τ^2^ and I^2^), to evaluate the influence of individual studies on both the pooled estimate and heterogeneity (33). Studies were considered potentially influential when omitting them resulted in a meaningful change in the direction, statistical significance, or magnitude of the pooled effect, or produced notable shifts in τ^2^ and/or I^2^ (33). Full LOO outputs were tabulated and provided in the Supplementary material.

Assessment of publication bias and small-study effects was conducted only when the minimum evidence threshold was met. For meta-analyses including at least 10 studies (*k* ≥ 10), we first inspected funnel plots to visually evaluate the symmetry of effect-size dispersion (34). We then applied Egger's regression test to formally examine funnel plot asymmetry as an indicator of potential small-study effects or publication bias (35). Given the limited statistical power of asymmetry tests in sparse meta-analyses, we did not perform publication-bias diagnostics for analytic units with fewer than 10 studies (36). All funnel plots and corresponding Egger test results are reported in the Supplementary material.

### 2.11 Software and significance threshold

Analyses were performed in Stata 16.0 (StataCorp, College Station, TX, United States) using the meta, metareg/metan, and parmest suites (full do-files are provided in the Supplementary material). Two-sided statistical significance was set at *P* < 0.05 unless otherwise noted. We report *P-values*, 95% CIs, τ^2^, I^2^, and R^2^_analog to facilitate interpretation.

## 3 Results

### 3.1 Study selection and characteristics

A total of 23 studies met the inclusion criteria (PRISMA diagram in Figure 1; full characteristics in Table 1). These studies spanned a diverse set of instant-messaging platforms, including WhatsApp, WeChat, LINE, Facebook Messenger, Telegram, KakaoTalk, and Naver Band. Intervention formats ranged from unidirectional push messaging to interactive group chats and hybrid models. Follow-up duration and intervention intensity varied widely across studies, as did measurement instruments. Six pre-specified outcome domains were examined: behavior, knowledge, attitudes, other clinical outcomes, complications, and glycemic outcomes.

### 3.2 Pooled effects by outcome domain

To enhance interpretability and avoid the mixture of outcomes of different natures, the main pooled analyses of this study were conducted, respectively within six preset node domains. Continuous outcomes were combined using standardized mean difference (SMD). Complication outcomes were synthesized using risk ratios (RRs).

**Figure 2:**
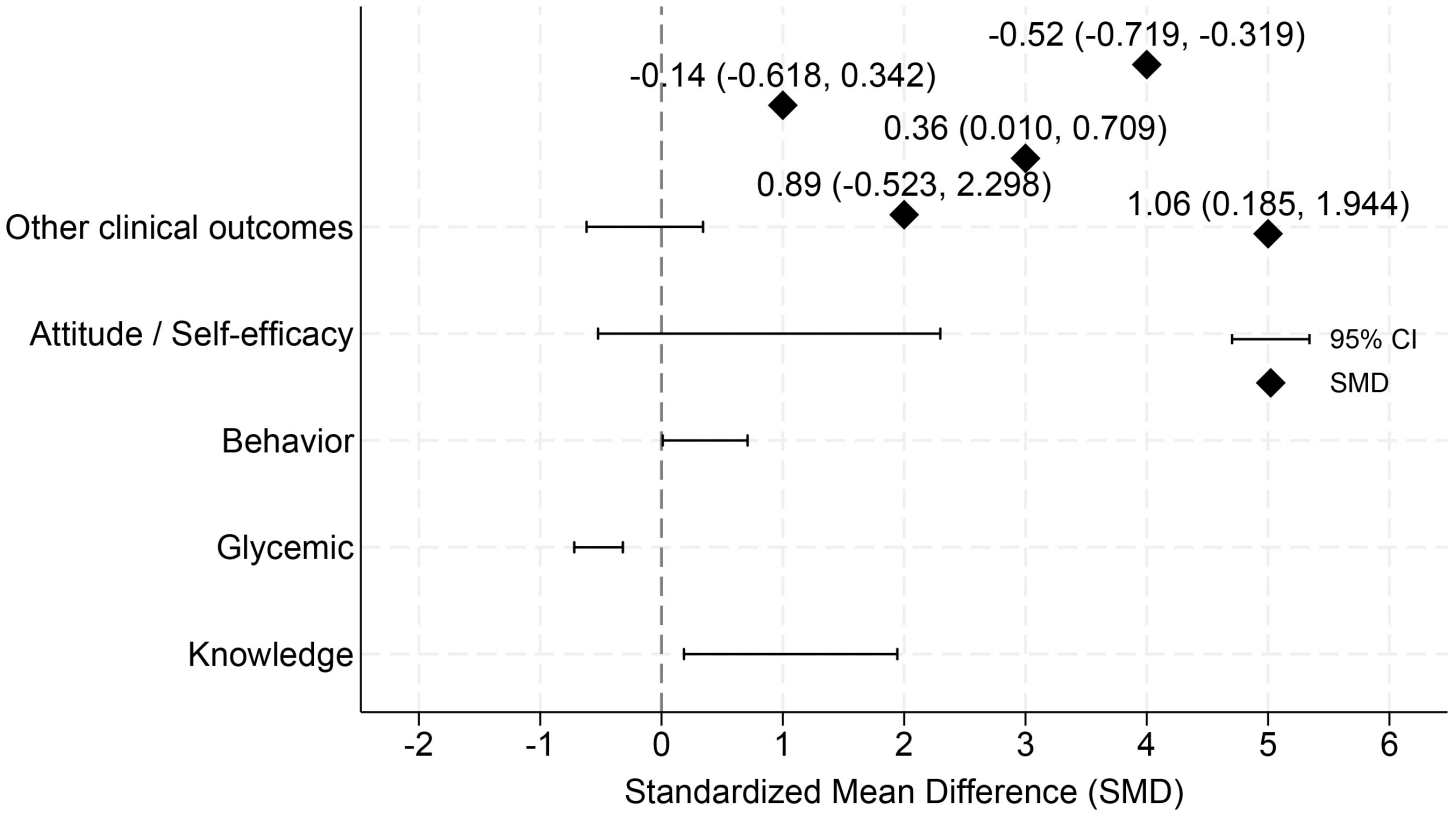
Overview of pooled standardized effects across five outcome domains.

As shown in Figure 2, we synthesized continuous outcomes across the included studies by pre-specified outcome domains and estimated pooled standardized mean differences (SMDs) with 95% confidence intervals using a random-effects model (REML). To provide an overall overview of continuous outcomes, Figure 2 summarizes five domains: diabetes-related knowledge, glycemic control, behavior, attitude/self-efficacy, and other clinical outcomes. Among these domains, diabetes-related knowledge demonstrated the largest pooled effect, suggesting that instant messaging–based interventions may substantially improve patients' diabetes-related knowledge (SMD = 1.065, 95% CI: 0.185–1.944). The outcome of blood glucose control also showed stable and clinically significant improvement effects (SMD = −0.519, 95% CI: −0.719 to −0.319; a negative value indicates a decrease in blood glucose level, which is a favorable direction). Health behavior outcomes showed small to moderate significant improvements (SMD = 0.359, 95% CI: 0.010–0.709).

In contrast, although the combined effect direction of the attitude/self-efficacy outcome was positive, the confidence interval was relatively wide and did not reach statistical significance (SMD = 0.887, 95% CI: −0.523 to 2.298), suggesting that there was considerable uncertainty in the research results. Other clinical outcomes (such as body weight, blood pressure, blood lipid, etc.) did not show significant improvement overall (SMD = −0.138, 95% CI: −0.618 to 0.342).

**Figure 3:**
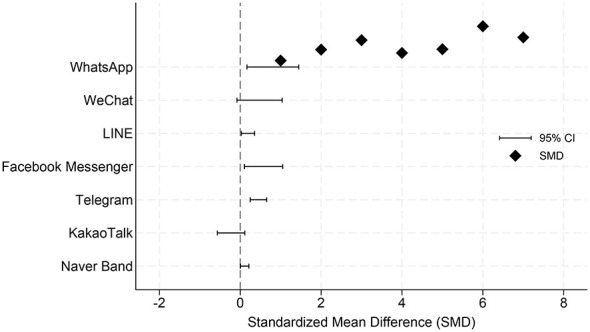
Platform-level pooled effects across continuous outcomes.

For diabetes-related complication outcomes, the pooled estimate suggested a potential reduction in complication risk (RR = 0.67, 95% CI 0.45–1.00). However, the confidence interval included the null value and the evidence was based on a small number of studies, so the result should be interpreted cautiously. Complication outcomes were not included in Figures 2, 3 because they were reported as binary endpoints (risk ratios/odds ratios) rather than continuous measures and were therefore synthesized separately using log-transformed effect estimates. Forest plots for the six outcome domains are provided in Supplementary Figures S1–S6.

Figure 2 presents the pooled standardized mean differences across five outcome domains using random-effects models. Improvements were observed in knowledge, behavioral outcomes, and glycemic control, whereas effects on attitudes/self-efficacy and other clinical outcomes were more heterogeneous and did not reach statistical significance. This platform-level synthesis pools continuous outcomes across outcome domains and is intended to describe overall patterns rather than domain-specific effects.

### 3.3 Platform-level pooled effects across outcomes

This analysis represents an overall meta-analysis across outcome domains, aiming to describe the overall effect distribution of studies on different platforms; key conclusions are still based on the stratified results by outcome domains. A random-effects model (REML) was used to conduct a platform-level meta-analysis of the intervention effects of different instant messaging platforms without distinguishing specific outcome domains (see Figure 3). The results showed that the overall effect size of the WhatsApp platform was small but statistically significant (SMD = 0.108, 95% CI: 0.004–0.213; *k* = 8). The overall combined effect of the WeChat platform did not reach statistical significance, with its confidence interval crossing zero (SMD = −0.227, 95% CI: −0.567–0.113; *k* = 5). The LINE platform demonstrated a moderate and statistically significant combined effect (SMD = 0.464, 95% CI: 0.247–0.654; *k* = 3). The Facebook Messenger platform also showed a significant overall effect (SMD = 0.577, 95% CI: 0.103–1.051; *k* = 4). The Telegram platform had a smaller overall effect size but still reached statistical significance (SMD = 0.191, 95% CI: 0.027–0.355; *k* = 4). The Naver Band platform had the largest combined effect estimate (SMD = 0.806, 95% CI: 0.165–1.447; *k* = 4), but its confidence interval was relatively wide.

Figure 3 presents platform-level pooled effects for continuous outcomes estimated using random-effects models (REML). When aggregating all eligible continuous outcomes across domains, heterogeneity in pooled effects was observed across platforms. Interventions delivered via Naver Band, Facebook Messenger, LINE, Telegram, and KakaoTalk were associated with positive pooled effects, whereas the pooled estimate for WeChat crossed the null value. WhatsApp showed a small but statistically significant pooled effect (SMD = 0.108, 95% CI: 0.004–0.213; *k* = 8). These platform-level estimates provide a high-level overview of between-platform variation but do not account for differences across outcome domains. It should be noted that the overall effect at the platform level mentioned above is a combined result across different outcome domains, mainly used to describe the overall distribution characteristics of the intervention effects of different platforms, and does not represent the comparison results among platforms within a single outcome domain.

### 3.4 Platform stratified analysis results

Platform-stratified analyses were reported only when a given platform contributed at least three effect sizes within a specific outcome domain (*k* ≥ 3). In the glycemic-control domain, WeChat-based studies showed a larger and more consistent improvement (SMD = −0.734, 95% CI −0.980 to −0.487), whereas the pooled effect for WhatsApp was smaller and not statistically significant (SMD = −0.217, 95% CI −0.701 to 0.268). In the health-behavior domain, WhatsApp yielded the largest pooled benefit (SMD = 0.754, 95% CI 0.363 to 1.145), while the corresponding estimate for WeChat was smaller and non-significant (SMD = 0.121, 95% CI −0.536 to 0.779). For other clinical outcomes, WeChat-based interventions showed a significant improvement (SMD = −0.467, 95% CI −0.744 to −0.190), whereas the WhatsApp estimate was highly unstable, with a very wide confidence interval (SMD = 0.830, 95% CI −1.090 to 2.750), indicating substantial imprecision.

Platform-stratified analyses were not performed for complication outcomes because no platform contributed ≥3 eligible effect sizes in that domain. Overall, these findings suggest clear outcome-domain dependence in platform-specific pooled effects: certain platforms appear to show more consistent signals in particular domains, but the observed between-platform differences likely reflect heterogeneity in intervention design and implementation (e.g., interactivity, dosing, feedback, and integration with care) rather than intrinsic platform properties. The complete platform-by-domain pooled estimates are summarized in Table 2.

**Table 2:** Platform-stratified pooled effects by outcome domain.

**Outcome domain**	**Platform**	***k***	**Pooled effect**	**95% CI**	***I^2^* (%)**
Glycemic	WeChat	11	−0.734	−0.980 to −0.487	85.2
	WhatsApp	3	−0.217	−0.701 to 0.268	78.0
	Facebook	2	NE	—	—
	LINE	1	NE	—	—
	Telegram	1	NE	—	—
	Naver Band	1	NE	—	—
Behavior	WhatsApp	5	0.754	0.363 to 1.145	78.3
	WeChat	8	0.121	−0.536 to 0.779	96.0
	Facebook	1	NE	—	—
	LINE	1	NE	—	—
	Telegram	1	NE	—	—
	Naver Band	1	NE	—	—
Other clinical	WeChat	7	−0.467	−0.744 to −0.190	96.0
	WhatsApp	3	0.830	−1.090 to 2.750	—†
	Facebook	1	NE	—	—
	LINE	0	NE	—	—
	Telegram	0	NE	—	—
	Naver Band	0	NE	—	—
Attitude/self-efficacy	WeChat	1	NE	—	—
	WhatsApp	3	0.887	−0.523 to 2.298	97.3
	Other platforms	< 3 each	NE	—	—
Complication (binary)	WeChat	2	NE	—	—
	WhatsApp	1	NE	—	—
	Other platforms	0	NE	—	—
Knowledge	WeChat	4	1.001	0.081 to 1.920	97.6
	WhatsApp	2	NE	—	—
	LINE	2	NE	—	—
	Other platforms	< 3	NE	—	—

This table reports pooled effects estimated using random-effects models with restricted maximum likelihood (REML), stratified by outcome domain × platform. To avoid unstable estimates arising from sparse strata, pooled effects are reported only when a given platform contributes k ≥ 3 effect sizes within a specific outcome domain; all other combinations are labeled as NE (not estimable). Here, k denotes the number of effect sizes contributing to the pooled estimate rather than the number of trials. I^2^ represents the proportion of between-study heterogeneity; when I^2^ was not available or not reported due to estimation instability, it is indicated by “—” †.

Direction of effect: Continuous outcomes are summarized using standardized mean differences (SMDs), where positive values generally indicate improvement; for glycemic outcomes, negative values indicate reductions in glucose levels and thus represent a favorable direction. Complication outcomes are binary and were synthesized using risk ratios (RRs) or their logarithmic form (logRR); strata corresponding to complication outcomes should therefore be interpreted on the binary outcome scale.

### 3.5 Sensitivity analysis

Leave-one-out (LOO) sensitivity analyses were conducted for all reportable meta-analytic models. Overall, sequentially omitting one study at a time did not materially alter the direction of the pooled effects within each outcome domain, indicating that the main conclusions were robust and not driven by any single trial.

In the diabetes-knowledge domain, the re-estimated pooled effect (θ) ranged from 0.66 to 1.21 across LOO iterations, with a maximum absolute deviation of 0.41 from the full-sample estimate. In the glycemic-control domain, θ varied narrowly from −0.56 to −0.46, with a maximum change of 0.055, suggesting particularly high stability. Similarly, in the health-behavior domain, θ ranged from 0.27 to 0.44, with a maximum change of 0.09. Importantly, for these three domains (knowledge, glycemic control, and behavior), the direction of effect and the overall statistical interpretation remained unchanged across all LOO models.

By contrast, larger fluctuations were observed in the attitude/self-efficacy domain and in the complications domain, indicating greater sensitivity to individual studies. Specifically, the attitude/self-efficacy pooled effect ranged from 0.35 to 1.50, and the pooled log risk ratio for complications ranged from −0.44 to 0.51 across LOO iterations. Nevertheless, despite this increased variability, the overall direction of the estimates did not show a substantive reversal. For other clinical outcomes, variability was moderate (θ range: −0.34 to −0.05), and the corresponding inferential conclusions remained consistent. Full LOO results, including the pooled estimates and 95% confidence intervals for each omission, are provided in the Supplementary material (LOO_^*^.csv).

### 3.6 Meta-regression analysis: sample size and follow-up time

To assess whether study-level characteristics contributed to between-study variation in effect sizes, we performed exploratory random-effects meta-regression analyses using follow-up duration and sample size as pre-specified covariates. Follow-up duration was not significantly associated with the standardized effect size (β = 0.002, *p* = 0.598), and the model explained only a negligible proportion of the between-study variability (*R*^2^ = 0.013). Likewise, log-transformed sample size did not significantly predict effect magnitude. Overall, these findings provide no evidence of a linear association between effect size and either follow-up duration or study size; however, given the exploratory nature of these analyses and the limited explanatory power observed, the results should be interpreted with caution.

### 3.7 Evaluation of publication bias and small sample effects

For meta-analyses with at least 10 contributing studies, we assessed potential small-study effects by visually inspecting funnel plots and formally testing funnel plot asymmetry using Egger's regression. Across analytic units meeting these criteria, the funnel plots did not show clear systematic asymmetry, and Egger's tests provided no strong evidence of small-study effects. All funnel plots and corresponding Egger test results (including overall and stratified assessments) are presented in Supplementary Figures S7–S9.

### 3.8 Rik of bias

**Figure 4:**
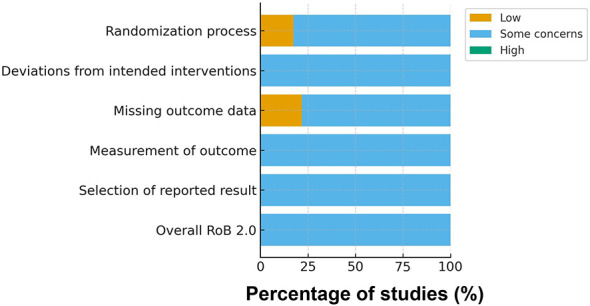
Risk of bias summary for included trials (Cochrane RoB 2.0).

**Figure 5:**
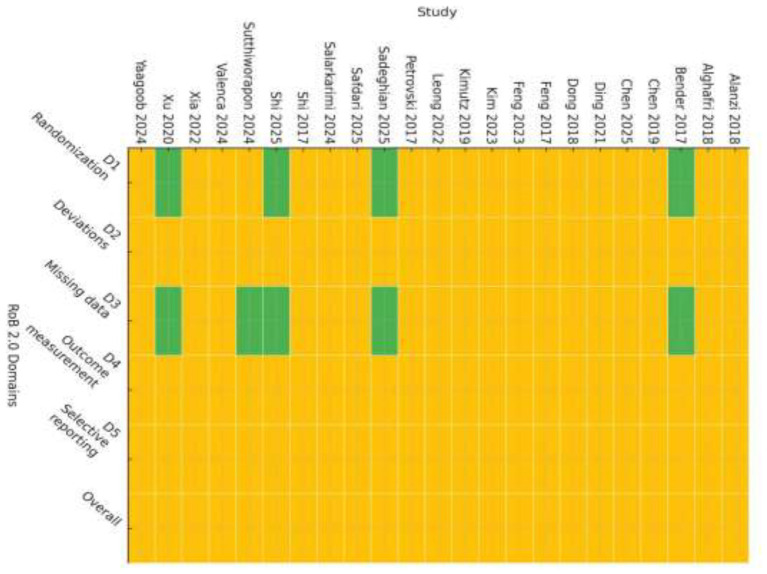
Risk of bias assessment (RoB 2.0 traffic-light plot).

Using the Cochrane Risk of Bias 2 (RoB 2) tool, all included trials were judged as having some concerns for overall risk of bias (23/23, 100%), reflecting common methodological challenges in messaging-based behavioral interventions. As shown in Figure 4, at the domain level, some concerns were consistently noted for deviations from intended interventions (D2), measurement of outcomes (D4), and selection of the reported result (D5), largely due to limited feasibility of blinding, frequent reliance on self-reported outcomes, and incomplete preregistration or insufficiently specified analytic plans in several trials. For the randomization process (D1), 4/23 (17.4%) trials were rated low risk and 19/23 (82.6%) as some concerns; for missing outcome data (D3), 5/23 (21.7%) were rated low risk and 18/23 (78.3%) as some concerns. A detailed study-level risk-of-bias assessment is presented in Figure 5, which illustrates the domain-specific judgments for each individual trial. Overall, most concerns arose from the inherent challenges of blinding in messaging-based interventions and the frequent reliance on self-reported behavioral outcomes. Detailed domain-level judgments are provided in Table 2.

## 4 Discussion

### 4.1 Summary of evidence

In this systematic review and meta-analysis, we synthesized evidence on instant-messaging–based interventions delivered through multiple platforms across six pre-specified outcome domains. Overall pooled effects were generally small to moderate and accompanied by substantial heterogeneity, indicating that a single global summary estimate provides limited explanatory value. To improve interpretability, we reorganized the evidence using a platform-sensitive and outcome-domain–structured approach.

At the platform level, the pooled “platform overview” of continuous outcomes (Figure 3) suggested variation in average effects across platforms. Telegram, LINE, and Facebook Messenger yielded positive pooled SMDs with confidence intervals that did not cross the null line, whereas WeChat showed a negative pooled estimate with a confidence interval crossing the null, and WhatsApp showed a small positive effect. Importantly, Figure 3 is intended as a descriptive overview that aggregates continuous outcomes across domains; it should be interpreted as illustrating broad patterns rather than substituting for domain-specific inferences.

At the outcome-domain level (Figure 2), behavioral outcomes showed the most consistent signals of benefit. Improvements were also observed for glycemic control and selected other clinical indicators in specific strata, whereas effects for attitudes/self-efficacy and other clinical outcomes were more heterogeneous and often did not reach statistical significance. For diabetes-related complications (binary outcomes), the pooled estimate suggested a potential reduction in risk; however, this finding was based on only three studies and remained imprecise, with the confidence interval approaching the null. Accordingly, complication results should be interpreted cautiously (see Supplementary material).

Across analyses, strata that combined highly heterogeneous intervention designs, mixed measurement constructs, or different outcome types tended to yield pooled effects close to zero with substantial between-study variability. Collectively, these findings suggest that observed effects are more likely driven by intervention design features and implementation context than by platform labels alone. Consistent with this interpretation, our exploratory meta-regression found no evidence that follow-up duration or study size explained meaningful variability in effect estimates, and overall explanatory power was low. These meta-regression analyses were exploratory and likely underpowered given the limited number of studies within strata; therefore, null moderator findings should be interpreted cautiously and not taken as evidence of no effect modification.

### 4.2 Methodological strengths and analytical approach

A core methodological contribution of this study is that we did not rely on a single global pooled estimate to judge the effectiveness of instant-messaging interventions. Instead, we adopted a platform-sensitive and outcome-domain–structured synthesis framework that organizes evidence in closer alignment with plausible clinical pathways and behavioral mechanisms. This approach is particularly relevant in digital health evidence synthesis, where platform labels, outcome constructs, and measurement instruments are often highly heterogeneous; under such conditions, global averages can obscure meaningful structural variation and limit implementation relevance.

Second, beyond reporting pooled effects and confidence intervals, we systematically presented τ^2^, I^2^, and prediction intervals, thereby extending uncertainty quantification from statistical significance to the range of effects that might reasonably be expected across future, comparable real-world settings. This is especially important for instant-messaging interventions, whose effects may be sensitive to context and delivery fidelity.

Third, we conducted exploratory random-effects meta-regression using follow-up duration and log-transformed sample size as pre-specified study-level moderators. These covariates explained little between-study variability, providing limited support for study scale or follow-up length as primary drivers of heterogeneity and motivating greater attention to actionable sources of variation—such as interactivity, dose/intensity, feedback design, and integration with care pathways.

Finally, we strengthened transparency and reproducibility through robustness checks (e.g., leave-one-out analyses) and small-study effect assessments, and we reported complete stratified estimates together with corresponding forest plots and summary tables in the Supplementary material, facilitating verification and future updating of the evidence base.

### 4.3 Theoretical integration: a design-centric interpretive framework

Integrating platform-level and outcome-domain–level findings, we propose a design-centric interpretation: the effectiveness of instant-messaging interventions appears to depend less on platform identity *per se* and more on whether intervention components are aligned with the mechanisms they aim to activate. Platforms provide accessibility and interaction affordances, but they are unlikely to constitute the “active ingredient.” Even within the same platform, trials varied substantially in interactivity, feedback frequency, clinical involvement, and implementation fidelity, underscoring the heterogeneity of design and delivery. Such design-level heterogeneity plausibly contributes to unstable pooled effects within platforms. Therefore, observed between-platform differences should be interpreted as reflecting clusters of design and implementation patterns rather than intrinsic superiority or inferiority of particular platforms; causal attribution to platform labels is not warranted. Moreover, platform choice is often geographically and institutionally patterned, such that apparent between-platform differences may partly reflect differences in healthcare systems, implementation settings, and participant characteristics rather than platform-specific effects.

Across outcome domains, behavioral outcomes showed the most consistent benefits, which aligns with instant messaging as a high-frequency, low-friction channel for reminders, reinforcement, feedback, and social support—mechanisms that are more readily captured by continuous behavioral measures. By contrast, distal clinical endpoints such as glycemic control typically require sustained behavior change, coordinated clinical decision-making, and, in many cases, treatment adjustment. When interventions lack explicit maintenance strategies (e.g., boosters, theme rotation, adaptive dosing) and are not embedded within monitoring–adjustment–escalation care pathways, proximal improvements may not translate reliably into durable clinical gains. The limited explanatory power of follow-up duration and study size in our exploratory meta-regression further suggests that key determinants of effect heterogeneity are more likely to reside in intervention design, implementation, and context rather than study scale or follow-up length alone. Importantly, the instant-messaging interventions synthesized in this review should not be interpreted as direct physician–patient clinical communication. In most included studies, messaging functions primarily served as automated reminders, structured feedback, peer support, or facilitation tools for intervention delivery, rather than channels for individualized clinical decision-making.

### 4.4 Main contributions

The contributions of this study can be summarized in three points. First, we advance a design-element–centered perspective, arguing that effectiveness is more likely driven by actionable components (e.g., interactivity, maintenance strategies, and integration with care pathways) than by platform labels. Second, by applying a platform-sensitive and outcome-domain–structured synthesis strategy, we move beyond global averaging and improve mechanistic interpretability and practice relevance. Third, we systematically reported uncertainty and robustness evidence and evaluated common study-level characteristics (follow-up duration and study size) as potential moderators, thereby narrowing the set of plausible explanations for heterogeneity and motivating future work to focus on design and implementation factors.

### 4.5 Practice and design implications

These practice and design implications should be interpreted as guidance for intervention design and implementation, rather than as endorsement of any platform for direct clinical communication. Regulatory, privacy, and health-system constraints may limit how instant-messaging tools can be deployed in routine care. In this context, the practical implications of these findings emphasize a shift from “platform comparisons” to “design optimization.” These implications are intended to be design-oriented and hypothesis-generating, and should be tested and refined in pragmatic trials and implementation research.

Prioritize interactivity and feedback. Compared with one-way broadcasting, two-way communication, structured feedback, moderated group management, periodic Q&A, and brief clinician touchpoints may better activate self-management and social reinforcement mechanisms. Here, “clinician touchpoints” refers to limited, protocolized involvement delivered through compliant channels (e.g., periodic review, structured Q&A, or escalation support), rather than individualized clinical messaging on public or non-secure social media platforms.

Design explicitly for maintenance. Incorporating maintenance strategies (e.g., booster sessions/messages, theme rotation, adaptive cadence, and individualized dosing) may help mitigate engagement fatigue and attenuate effect decay over time.

Embed interventions within care pathways. Linking messaging to monitoring–adjustment–escalation processes (e.g., remote monitoring, medication-adjustment rules, and referral thresholds) may increase the likelihood of durable improvement in distal clinical outcomes. Notably, few included trials explicitly integrated messaging into monitoring–adjustment–escalation pathways or medication titration protocols; this represents a key direction for future trials and implementation studies.

Measure proximal mediators and process indicators. Routine reporting of engagement, interaction intensity, perceived support, and self-efficacy would facilitate mechanism-focused interpretation and iterative optimization and improve the quality of future evidence synthesis.

### 4.6 Strengths and limitations

Key strengths of this study include preregistration, a platform-sensitive and outcome-domain–structured synthesis approach that improves interpretability, and decision-relevant uncertainty reporting through τ^2^, I^2^, and prediction intervals. We further strengthened credibility via leave-one-out sensitivity analyses and small-study effect assessments. Nonetheless, important limitations remain. First, residual heterogeneity persisted even after stratification, indicating that effects are likely shaped by design and implementation features that were not consistently measured or reported (e.g., interactivity, personalization, delivery intensity, and integration with care pathways). Second, several platform-by-domain subgroups contained few effect sizes, reducing statistical power and the precision of subgroup estimates. Third, while we did not observe clear evidence of small-study effects in analyses meeting minimum study thresholds, these assessments were not possible for all strata, and small-study bias cannot be definitively ruled out for continuous outcomes. In addition, by restricting inclusion to randomized controlled trials, this review prioritized internal validity and causal interpretability but may underrepresent real-world implementation dynamics. While inclusion of non-randomized studies could offer complementary insights into routine practice, such evidence also poses substantial challenges related to residual confounding and heterogeneity.

Finally, suboptimal reporting of intervention components and exposure metrics in primary trials limited more granular component analyses and precluded strong causal attribution regarding specific “active ingredients.”

## 5 Conclusion

Instant-messaging interventions show promising signals of benefit for behavioral outcomes and selected clinical indicators; however, the magnitude and consistency of effects appear highly contingent on intervention design, maintenance strategies, and implementation context. Accordingly, the key question is not whether a specific platform “works,” but which components—delivered at what intensity and for how long—and embedded within which care pathways are most likely to produce durable behavioral and clinical gains. By structuring the evidence by platform and outcome domain and systematically reporting uncertainty and robustness, this review offers a design-oriented evidence base to support implementation decisions and the optimization of future instant-messaging interventions.

## Data Availability

The original contributions presented in the study are included in the article/Supplementary material, further inquiries can be directed to the corresponding author.
