# Supplementary figures and images for: Instant messaging-based digital health interventions for diabetes management: a domain-structured systematic review and meta-analysis of randomized controlled trials
Source: Front Public Health. 2026 Mar 9;14:1780625. doi: 10.3389/fpubh.2026.1780625 (PMC13006411; doi:10.3389/fpubh.2026.1780625)

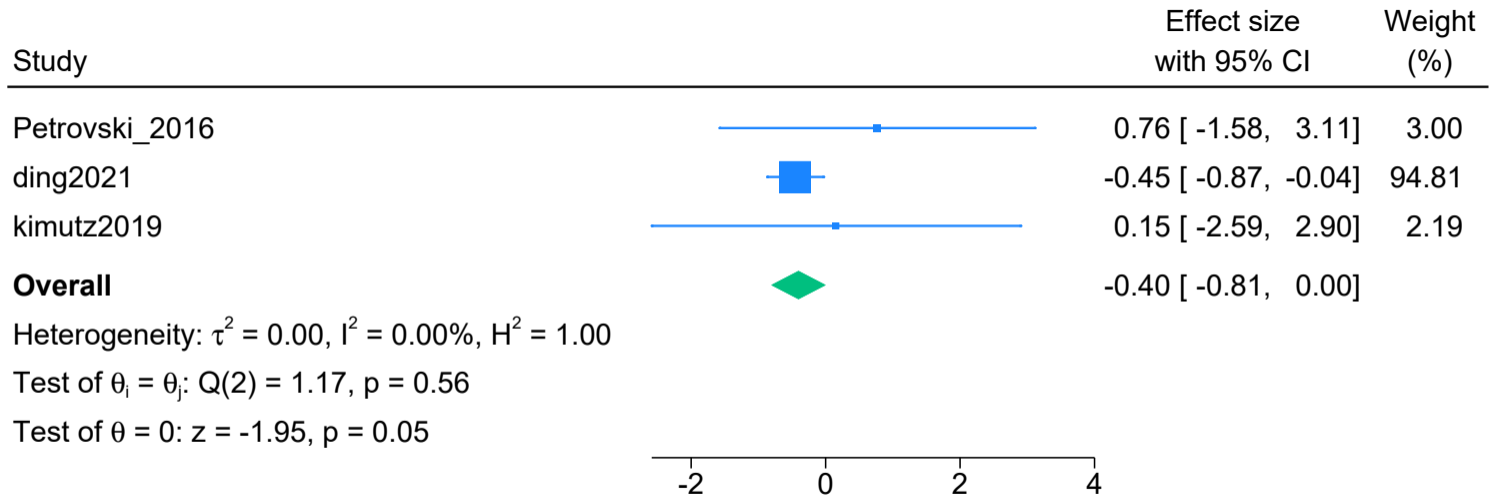

Random-effects REML model

Supplement: Supplementary file 2 [file Data_Sheet_1.pdf]

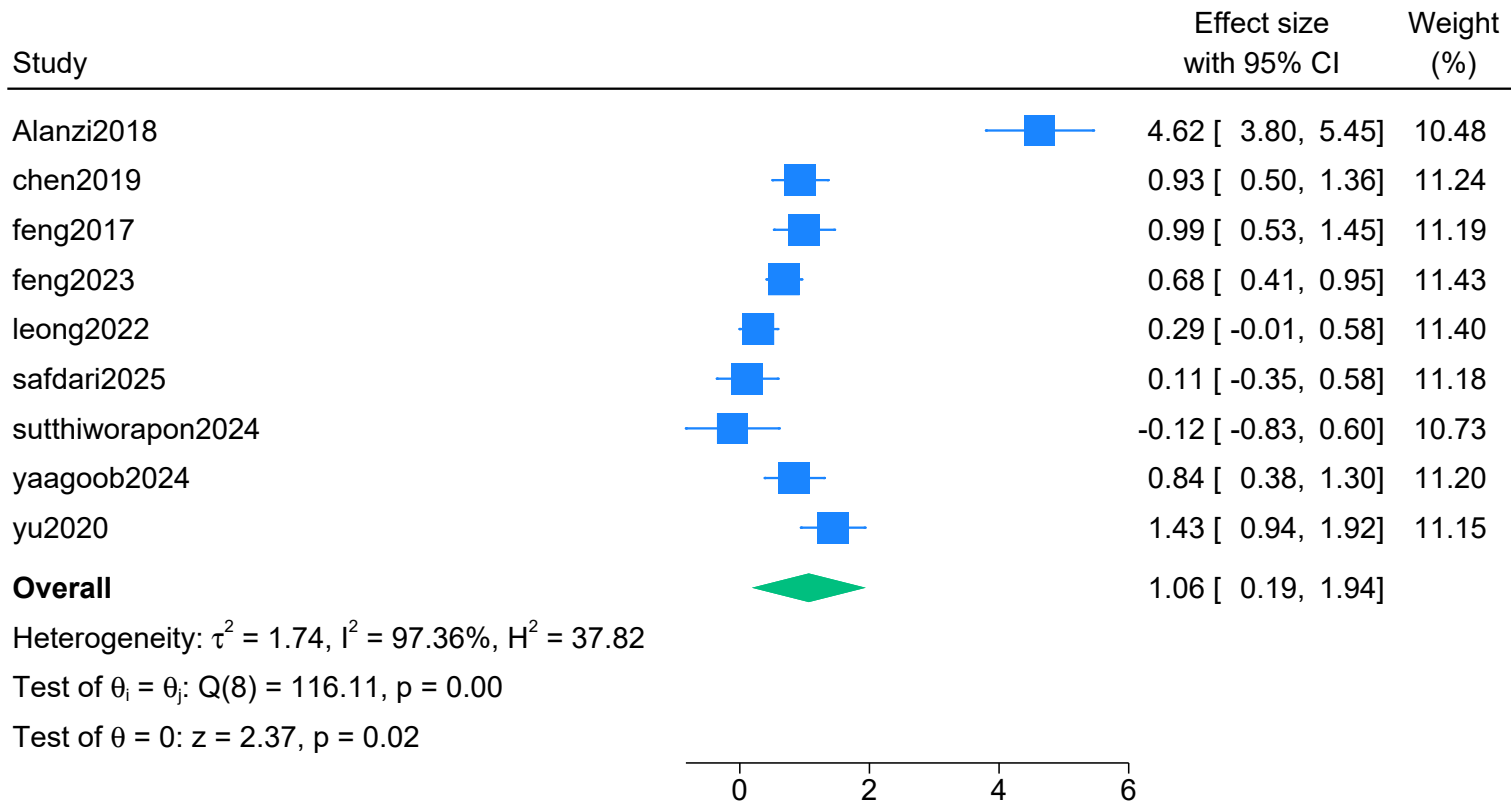

Supplement: Supplementary file 3 [file Data_Sheet_2.pdf]

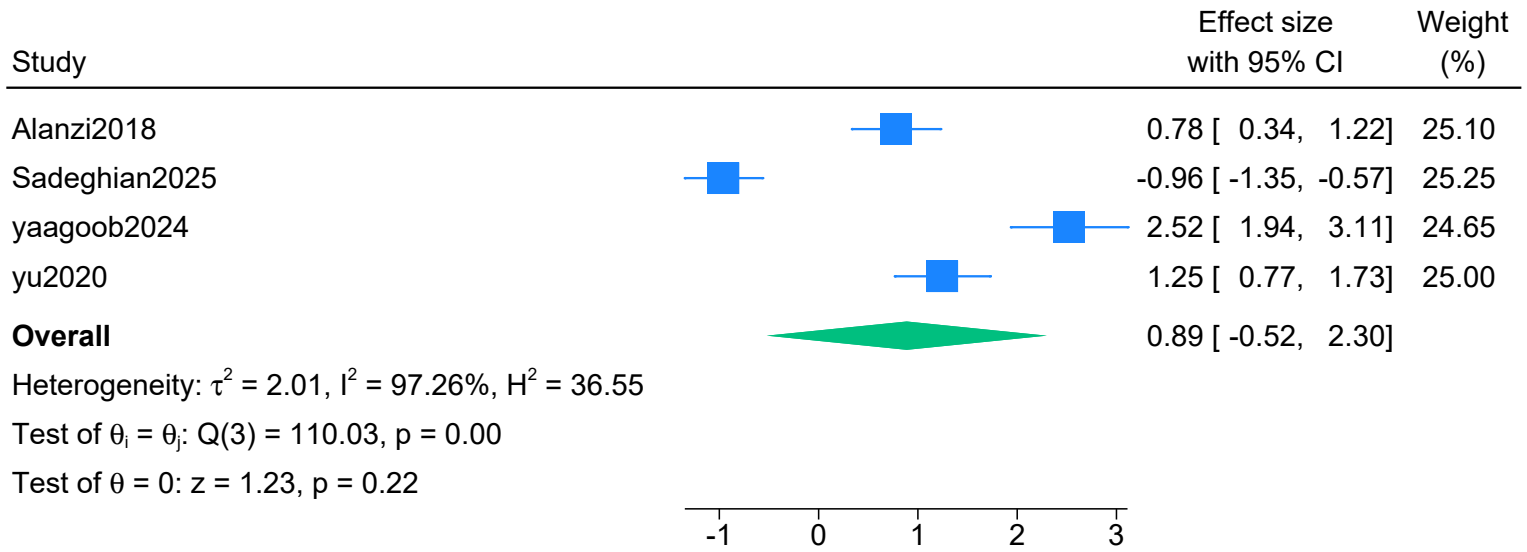

Random-effects REML model

Supplement: Supplementary file 4 [file Data_Sheet_3.pdf]

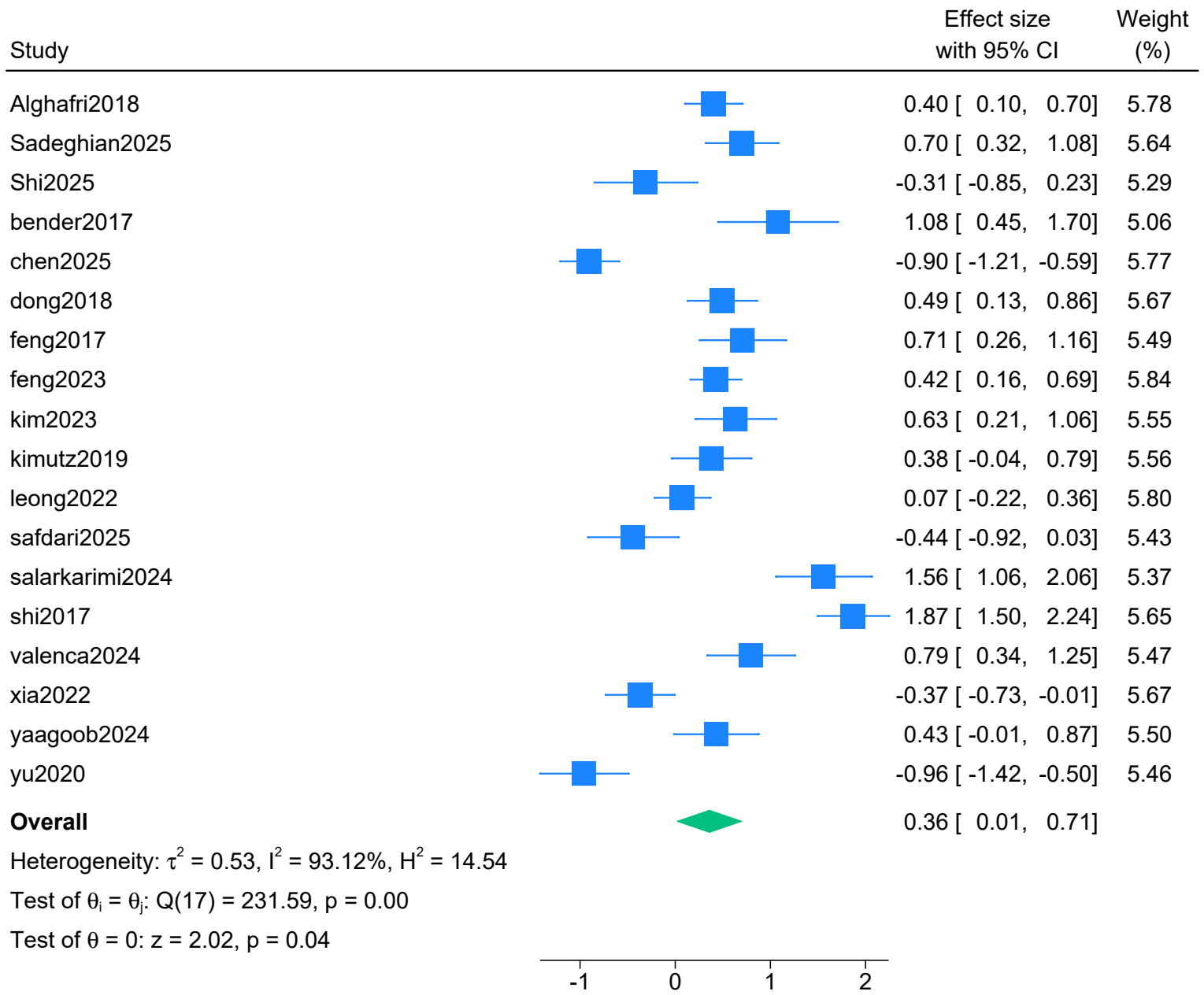

Random-effects REML model

Supplement: Supplementary file 5 [file Data_Sheet_4.pdf]

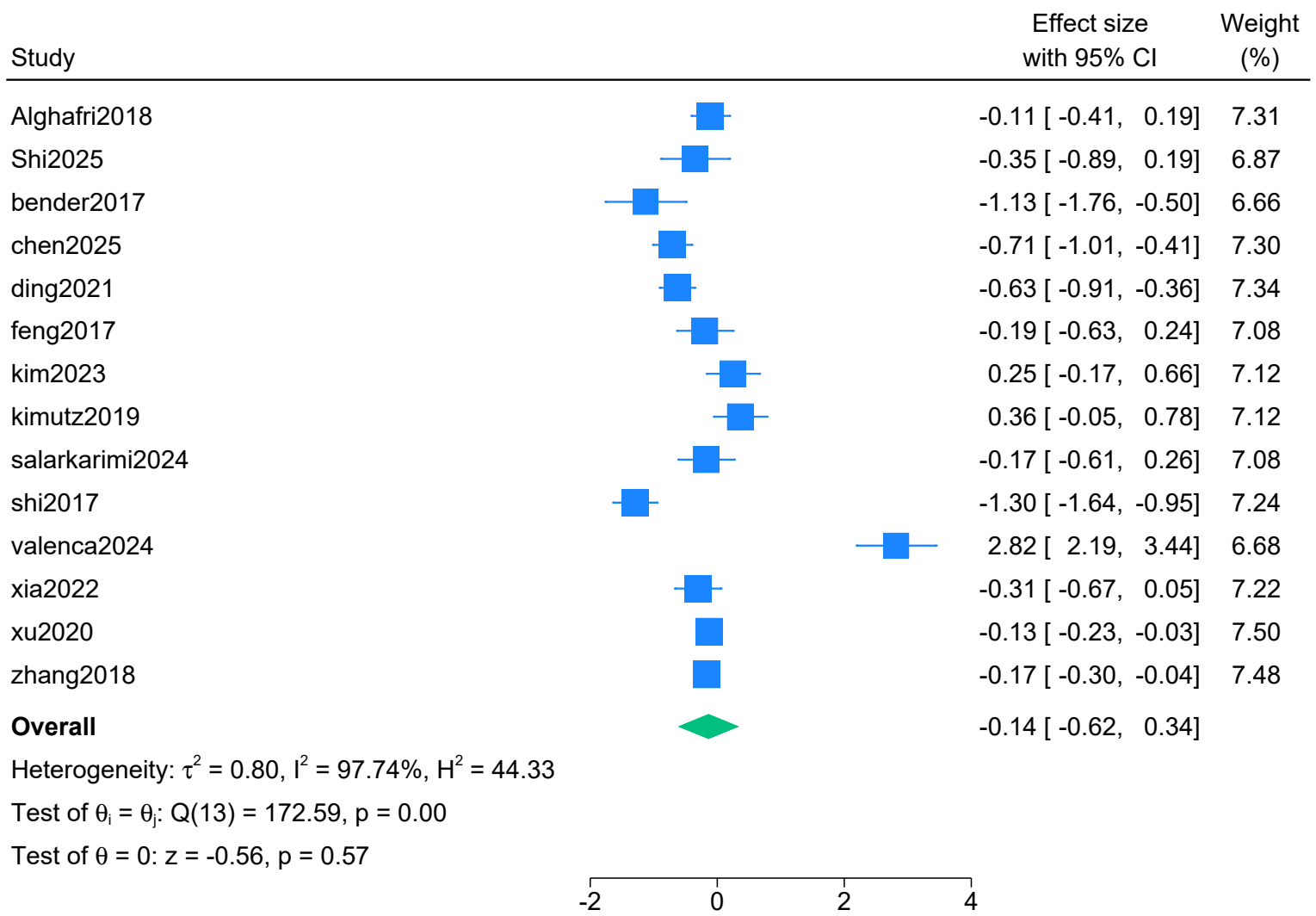

Random-effects REML model

Supplement: Supplementary file 6 [file Data_Sheet_5.pdf]

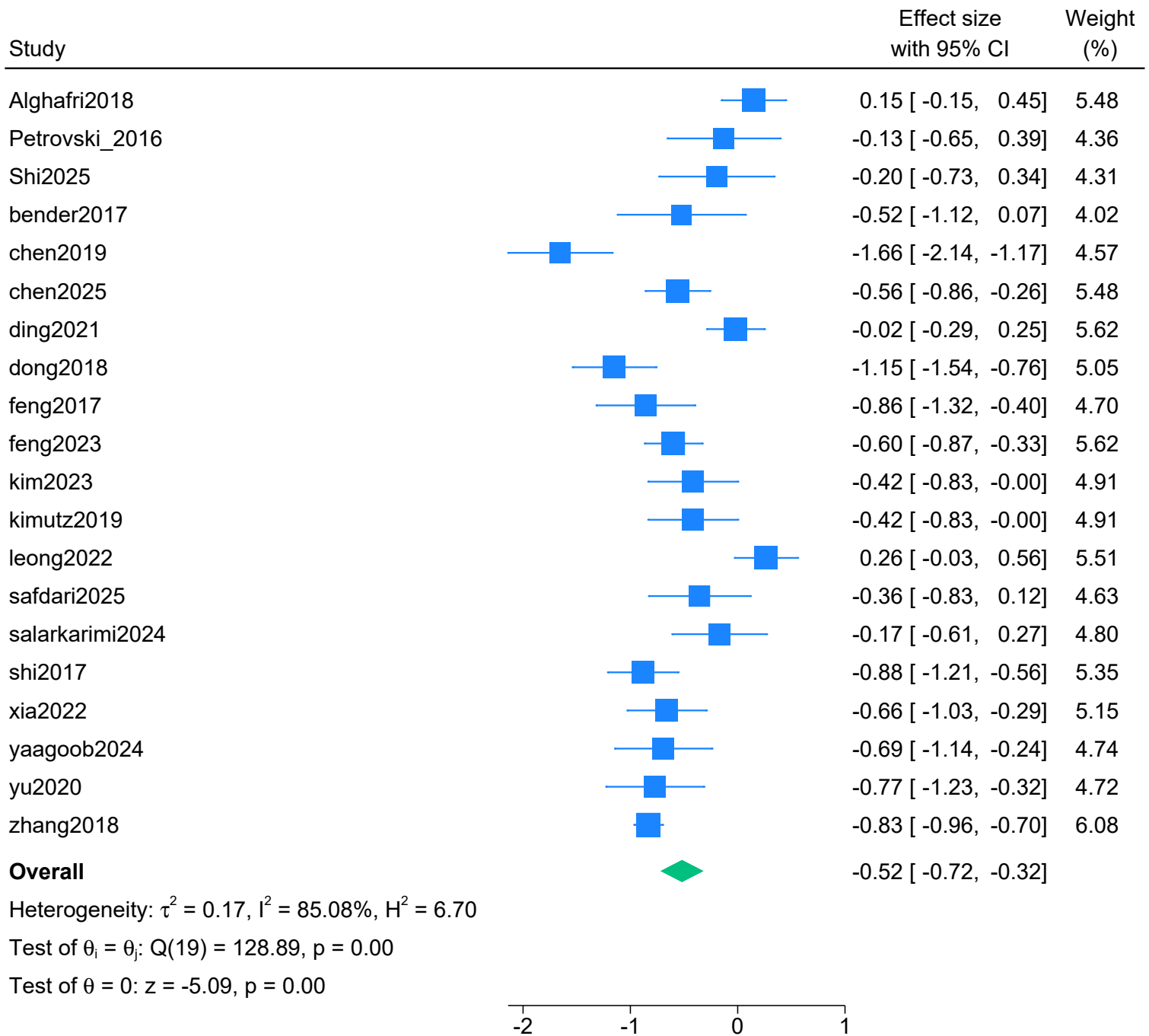

Supplement: Supplementary file 7 [file Data_Sheet_6.pdf]

# Funnel plot

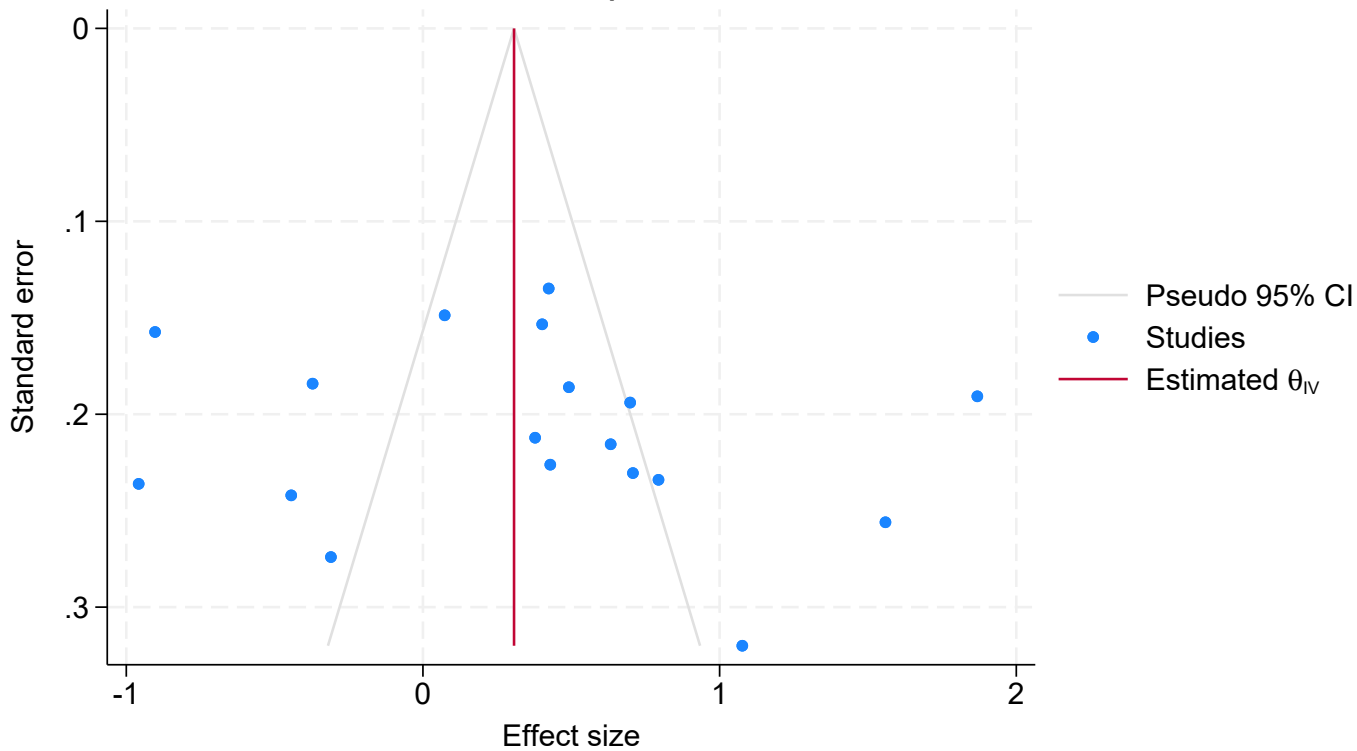

Supplement: Supplementary file 8 [file Data_Sheet_7.pdf]

# Funnel plot

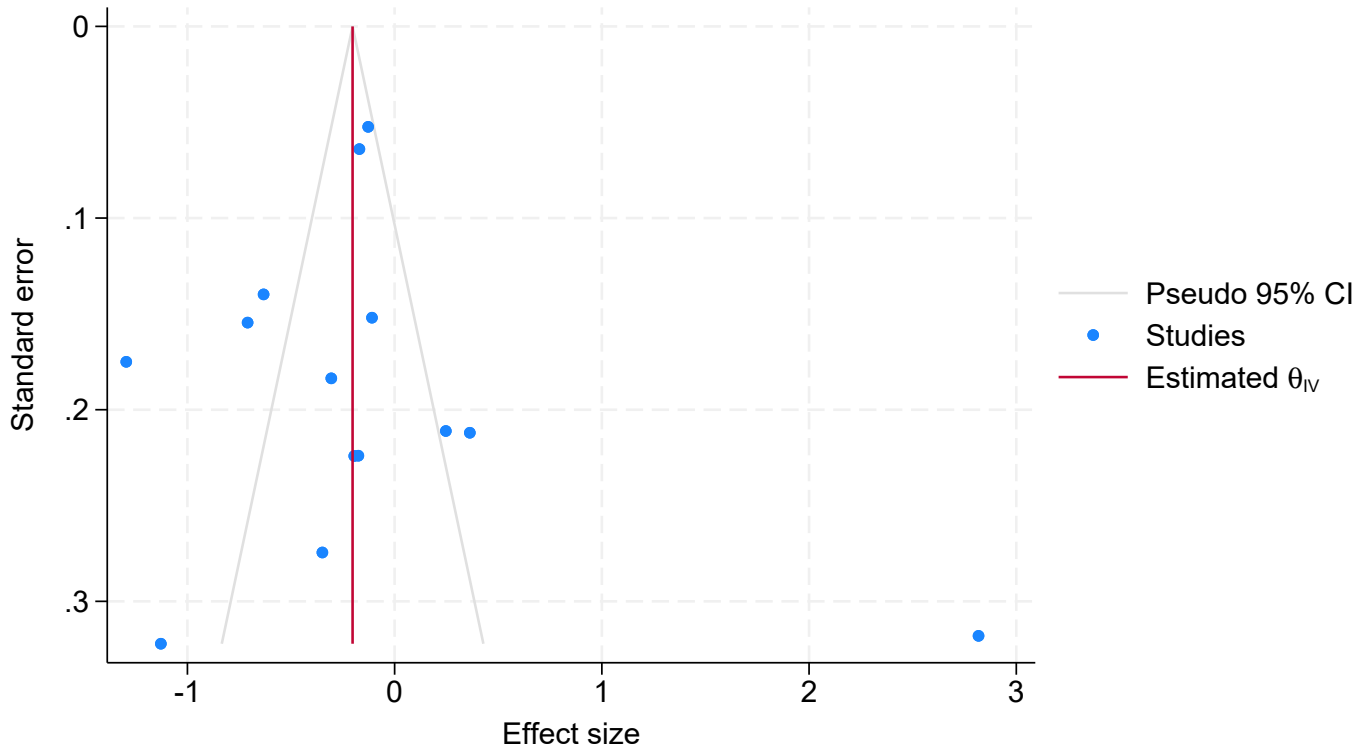

Supplement: Supplementary file 9 [file Data_Sheet_8.pdf]

# Funnel plot

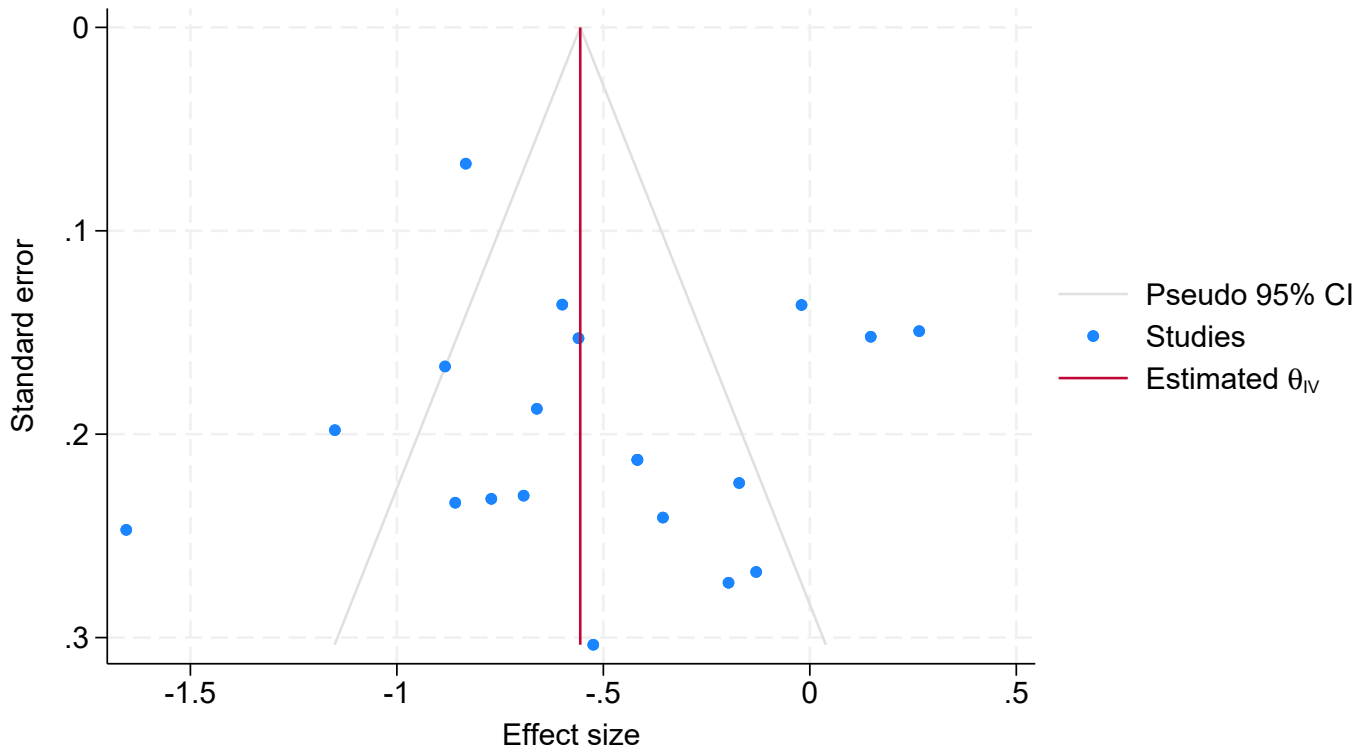

Supplement: Supplementary file 10 [file Data_Sheet_9.pdf]
